# Cortical ribbon sign on neuroimaging in a patient with hepatic encephalopathy secondary to herbal medicine usage

**DOI:** 10.31744/einstein_journal/2023AI0538

**Published:** 2023-10-26

**Authors:** Gabriel de Deus Vieira, Mariana Moreira Soares de Sá, Arthur de Medeiros Dias, Rafael Gemaque Lima Bentes, Augusto Celso Scarparo Amato, André Augusto Lemos Vidal de Negreiros, Ana Carolina Amaral de Andrade, Simone Reges Perales, Elaine Cristina de Ataide, Alexandre Foratto, Alfredo Damasceno

**Affiliations:** 1 Department of Neurology Universidade Estadual de Campinas Campinas SP Brazil Department of Neurology , Universidade Estadual de Campinas , Campinas , SP , Brazil .; 2 Department of Neuroradiology Universidade Estadual de Campinas Campinas SP Brazil Department of Neuroradiology , Universidade Estadual de Campinas , Campinas , SP , Brazil .; 3 Department of Digestive System Surgery Universidade Estadual de Campinas Campinas SP Brazil Department of Digestive System Surgery , Universidade Estadual de Campinas , Campinas , SP , Brazil .

The use of herbal medicines is growing exponentially despite the lack of scientific evidence regarding their effectivity and toxicity. ^( 1 , 2 )^ Moreover, the use of herbal medicines have been reported to be common in patients with chronic liver disease (30–62%); these medicines can lead to hepatotoxicity and serious hepatic side effects. ^( 3 )^ Hepatic encephalopathy is a complication of acute liver failure and chronic liver disease that causes cognitive dysfunction, motor deficits, and seizures. ^( 4 , 5 )^ Gastrointestinal hemorrhage, infection, dehydration, constipation, and use of medications are the most common precipitating factors. ^( 5 )^

**Figure 1:**
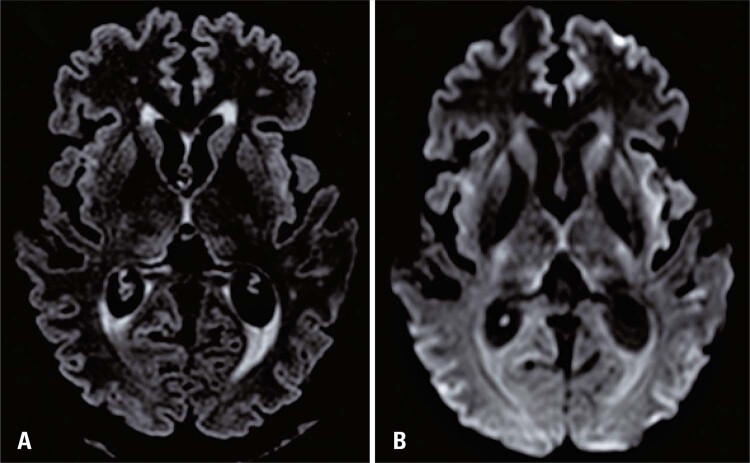
Axial magnetic resonance imaging. (A) FLAIR imaging sequence with hyperintensity involving practically the entire cortical ribbon; (B) Diffusion-weighted imaging sequence with diffusion restriction throughout the cerebral cortex, notably in the bilateral insulae

**Figure 2:**
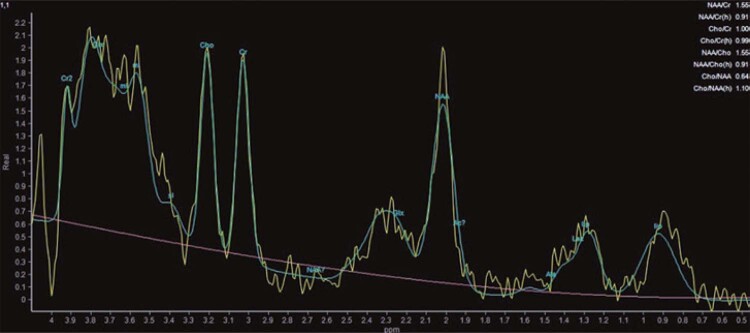
Proton magnetic resonance spectroscopy positioned in the right insular cortical region, showing glutamate/glutamine peak (increased in hepatic encephalopathy), slight decrease in myo-inosital (glial marker), and lipid/lactate peak (marker of necrosis/anaerobiosis)

This study reports the case of a previously healthy 37-year-old woman who developed acute liver failure and hepatic encephalopathy (international normalized ratio 3.5, total bilirubin 30.2mg/dL, direct bilirubin 15.5mg/dL, aspartate aminotransferase 423U/L, and alanine aminotransferase 459U/L) after consuming an herbal medicine ( *Citrus sinensis* ) for lose weight. The patient went to the hospital due to jaundice and malaise, being hospitalized for evaluation. She underwent brain magnetic resonance imaging (MRI) due to lethargy, revealing cortical ribbon sign in diffusion-weighted and fluid-attenuated inversion recovery (FLAIR) sequences and hypersignal in the caudate nuclei, putamen, and insula bilaterally ( Figure 1 ). The cortical ribbon sign is a typical finding in patients with Creutzfeldt–Jakob disease; however, it has also been reported in patients with infection, hypoxia, electrolyte derangements, and hepatic encephalopathy (as in the present case). ^( 6 , 7 )^ Moreover, magnetic resonance spectroscopy revealed an increase in glutamate/glutamine, typically observed in hepatic encephalopathy ( Figure 2 ). Owing to the severity of the condition, the patient underwent liver transplantation and is improving progressively.
